# Role of vascular function in predicting arteriovenous fistula outcomes: an observational pilot study

**DOI:** 10.1186/s40697-015-0055-8

**Published:** 2015-05-04

**Authors:** Jennifer M MacRae, Sofia Ahmed, Brenda Hemmelgarn, Yichun Sun, Billie-Jean Martin, Idan Roifman, Todd Anderson

**Affiliations:** Division of Nephrology, Faculty of Medicine, University of Calgary, Calgary, Canada; Department of Cardiac Sciences, Faculty of Medicine, University of Calgary, Calgary, Canada; Sunnybrook Health Sciences Centre, University of Toronto, Toronto, Canada

**Keywords:** Arteriovenous fistula, Endothelial function, Vascular reactivity, Peripheral arterial tonometry

## Abstract

**Background:**

Many arteriovenous fistula (AVF) fail prior to use due to lack of maturation or thrombosis. Determining vascular function prior to surgery may be helpful to predict subsequent AVF success. This is a feasibility study to describe the vascular function in a cohort of chronic kidney disease (CKD) patients who are awaiting AVF creation.

**Methods:**

A prospective cohort of 28 CKD patients expected to progress to HD underwent arterial stiffness (pulse wave velocity, PWV) and endothelial function testing (flow mediated dilation FMD, and peripheral arterial tonometry, PAT) one week prior to AVF creation. AVF success was defined as maintaining patency and achieving maturation. Post operative fistula assessment at 8 weeks evaluated maturation (clinical assessment of adequate fistula flowand ultrasound diameter ≥ 0.5 cm).

**Results:**

The median age 72 years (62 - 78), 75% males, eGFR 15 ml/min/1.73 m^2^ (12 – 18). 20 (71%) patients had successful AVF surgery with a mature AVF at 8 weeks. Patients with AVF success had higher mean PAT values 1.87 ± 0.52 than those with failed AVF 1.41 ± 0.24 p = 0.03.

**Conclusions:**

Microvascular endothelial function as measured using PAT may be useful as a predictor of AVF maturation and function. This simple non invasive marker of vascular function may be a useful tool to predict AVF outcomes.

## Abrégé

**Contexte:**

On attribue l’échec précoce de plusieurs fistules artérioveineuses (FAV) à un manque de maturation ou à une thrombose précoce. Une évaluation de la fonction vasculaire en phase préopératoire pourrait aider à prédire l’issue d’une FAV prochaine. Ceci est une étude de faisabilité de l’évaluation de la fonction vasculaire d’une cohorte de patients atteints d’insuffisance rénale chronique (IRC) en attente de confection de FAV.

**Méthode:**

L’étude de cohorte prospective comprenait 28 patients atteints d’une IRC évoluant vers des traitements d’hémodialyse. Une semaine avant la confection de la FAV, les participants ont subi des évaluations de la rigidité artérielle (analyse de l’onde de pouls) et de la fonction endothéliale (mesure de la vasodilatation médiée par le flux [*flow-mediated dilatation* ou FMD] et tonométrie artérielle périphérique ou EndoPAT) une semaine avant la confection de la FAV. Nous définissons le succès de la FAV comme le maintien de la perméabilité et l’atteinte de sa maturité. L’examen postopératoire de la fistule, 8 semaines après l’intervention, évaluait sa maturation (examen clinique du débit adéquat de la fistule, et évaluation du diamètre par ultrasons, soit ≥ 0,5 cm.).

**Résultats:**

L’âge moyen des participants était de 72 ans (62 – 78); 75% des participants étaient de sexe masculin. Le DFG estimé était de 15 ml/min/1,73 m2 (12 – 18). Le succès de la confection et la maturation de la FAV à 8 semaines ont été évalués auprès de 20 patients (71%). Les valeurs moyennes de l’EndoPAT étaient plus élevées chez les FAV complétées avec succès (1,87 ± 0,52), que chez les FAV en échec précoce (1,41 ± 0,24 p = 0,03). Après redressement de l’échantillon pour l’âge, le sexe et le DFG, l’EndoPAT était lié au succès de la FAV (pour chaque augmentation à l’EndoPAT, le taux de succès de la FAV était multiplié par 1,75; p = 0,02).

**Conclusions:**

La fonction microvasculaire endothéliale évaluée par EndoPAT pourrait être utilisée pour prédire la maturation et le fonctionnement d’une FAV. Cet indicateur simple et non invasif de la fonction vasculaire pourrait être également un outil pratique pour prédire le résultat de la confection d’une FAV.

## Background

Chronic kidney disease patients (CKD) have a large burden of vascular disease which contributes to their morbidity and leads to increased mortality. A thorough characterization of the vascular function of CKD patients may allow us to better predict the vascular outcomes in these patients. Once CKD patients progress to end stage renal disease, a functioning, reliable vascular access (VA) is crucial for the delivery of adequate hemodialysis (HD). A vascular access allows blood to be delivered to the dialysis machine and can be in the form of a fistula (AVF), graft or a central venous catheter.

Studies consistently demonstrate that AVF are the preferred type of VA as they provide the highest blood flow, suffer from the fewest complications [1] (such as stenosis or infection), and have the longest survival [2], and lowest associated mortality as compared to either catheters or grafts. Unfortunately anywhere from 30 to 61% of fistula will fail prior to their use for hemodialysis either due to lack of vessel maturation or spontaneous thrombosis [3]. A better understanding of the patient’s vasculature may help predict which patients are at highest risk of fistula failure.

A number of measures are available to assess vascular function and predict future cardiovascular risk such as pulse wave velocity (PWV) and flow mediated dilation (FMD) of the brachial artery. PWV characterizes arterial stiffness and is a strong independent predictor of cardiovascular mortality in both end-stage kidney patients [4] and the general population [5]. FMD provides an assessment of macrovascular endothelial cell function. Healthy endothelium releases a potent vasodilator (nitric oxide, NO) in response to ischemic stress such as cuff occlusion of the brachial artery. FMD is a measure of the change in the brachial artery diameter in response to cuff occlusion and subsequent NO release. Whereas FMD reflects the ability of a large artery to vasodilate, the subsequent change in blood flow that ensues represents the health of the peripheral resistance vessels or microvascular function. Microvascular endothelial function can be characterized by the velocity time integral (VTI) of the hyperemic velocity [6] or the pulse wave amplitude during reactive hyperemia. Peripheral arterial tonometry (PAT) [7,8] provides a measure of the pulse wave amplitude with reactive hyperemia, and when normalized to the baseline, it gives an index or a ratio with higher values representing better vascular health. Individuals with coronary artery disease (CAD) have been shown to have an impaired FMD response [9], a reduced VTI [6] and low PAT index [10]. These measures have also been shown to predict the development of cardiovascular disease in the general population [11] although literature amongst CKD patients is limited [12].

The primary objective of this study was twofold: to describe the arterial stiffness (PWV) and endothelial function markers (FMD, VTI and PAT) in a cohort of CKD patients referred for access creation and to determine the feasibility of using these vascular health markers as predictors of successful fistula maturation at 8 weeks post fistula creation. Secondary objectives were to determine if patient characteristics (age, diabetes status, CAD) were associated with successful AVF maturation and use. We hypothesized that measures of vascular health are associated with VA outcomes in the CKD population and that patients in whom an AVF was successfully created and matured would have better vascular health parameters than those in whom an AVF was unsuccessful.

## Methods

This was a prospective cohort study of CKD patients, 18 years or older, with an estimated glomerular filtration rate (eGFR) < 20 ml/min/1.73 m [2], who were expected to progress to hemodialysis and who had been referred for surgical assessment for possible AVF creation. Within this cohort there was a group of patients who were referred to the vascular access surgeons for planned fistula creation (and not just for a surgical assessment only). These patients received a date for planned AVF creation and are the focus of the current study. All patients, including those who were sent for a surgical assessment only, underwent vascular health testing; however amongst the patients with a planned AVF surgery this testing was done 1 week prior to the AVF creation date. Patients were excluded if they did not fast for 10 hours or had taken antihypertensive medication the morning of the testing. This study was approved by the University of Calgary Research Ethics Board and all subjects gave written informed consent. Demographic and clinical data were abstracted from the Southern Alberta Renal Program Database including patient characteristics such as age, gender, smoking status and comorbidities of diabetes mellitus (DM), CAD, peripheral vascular disease (PVD), congestive heart failure (CHF) and cerebrovascular disease (CVA).

### Vascular health testing

FMD, VTI, PWV, PAT and blood pressure were measured in the non-dominant arm one week prior to scheduled AVF creation. Patients fasted for at least 10 hours (including abstinence from caffeine, vasoactive medications, nicotine and alcohol) prior to testing. All studies were performed following a standardized procedure by a single operator at 23 C in the Endothelial Function Laboratory, Department of Cardiac Sciences, University of Calgary, Calgary, Canada. Patients rested for 5 minutes in a supine quiet position prior to blood pressure measurements (Welch Allyn Canada Ltd., Mississauga, ON) measured in duplicate. Baseline PWV measurements were taken using applanation tonometry (Sphygmacor Version 8,0, Atcor Medical), a non invasive pressure tonometer probe that was sequentially placed on the carotid and radial artery of the supine resting subject’s non-dominant arms. All PWV measurements were taken by the same person. The speed at which the pulse wave travels between the carotid and radial artery was determined using gated electrocardiographic data and translated into a pulse wave velocity. Augmentation index was calculated as the difference between the first and second aortic systolic peaks normalized to the pulse pressure [13].

FMD was determined using a high resolution ultrasound (SONOS 5500; Phillips Medical Systems, Andover, MA) equipped with 10 MHz linear array vascular transducers to measure the brachial artery diameter at baseline and with hyperemia. After baseline measurements of the brachial artery diameter and velocity were obtained, a blood pressure cuff was inflated to 200 mmHg or 50 mmHg above the systolic pressure on the distal portion of the non-dominant arm for 5 minutes to create distal limb ischemia. After release of the cuff, reactive hyperemia occurs and the flow in brachial artery increases. The peak brachial artery diameter for calculation of FMD was determined between 45 seconds and 2 minutes after cuff release. VTI was determined as the first full envelope after cuff release. Further details of FMD analysis are presented elsewhere [14]; FMD was calculated as: (hyperemic brachial artery diameter – baseline brachial artery diameter)/baseline diameter x 100%. All measurements of the FMD were obtained by one trained individual.

The PAT device (Itamar Medical, Caesaria, Israel) uses a pressure sensor to measure the arterial component of the fingertip volume changes that accompany the pulse wave. This fingertip plethysmograph is placed on the index finger of each hand for PAT assessment of the control (dominant arm) and ischemic limb (non dominant arm). The PAT signal is recorded continuously at baseline, throughout cuff occlusion and immediately after occlusion in order to give an index of the peak hyperemic fingertip volume to the baseline ratio normalized to the control arm. Measurements of PAT, VTI and FMD were taken simultaneously. Details of the PAT index are described elsewhere [7].

Biomarkers of vascular health (high sensitivity C reactive protein (hs-CRP), and parathyroid hormone (PTH) were obtained along with other laboratory values (hemoglobin, albumin, and creatinine)) at baseline coinciding with the time of vascular parameter measurement.

### Outcomes

Amongst CKD patients progressing to AVF creation the association between the vascular health parameters and AVF success at 8 weeks was explored. AVF success was defined as maintaining patency (thrill present) and achieving functional maturation. Post operative fistula assessment was done 8 weeks after creation to evaluate the maturation status of the fistula. Maturation was determined with ultrasound criteria of AVF diameter > 5 mm and adequate flow criteria assessed by the presence of a strong thrill at the anastamosis [15]. Maturation assessments done at 6 to 8 weeks post creation have been shown to predict the ability of the AVF to support dialysis with a mature AVF providing a functional AVF at dialysis start [15]. Dialysis suitability was defined as ability to support dialysis without the need for an alternate access in the first month of dialysis initiation.

### Statistical analysis

The primary outcome of this study was AVF success (patent and mature) at 8 weeks post creation. Baseline patient characteristics including age, gender and co-morbidities were compared between the patients with successful AVF and those with unsuccessful AVF. Categorical variables were represented by frequency (percentage) and comparisons were made via the Chi-square or Fisher’s Exact test. Underlying distributions of continuous variables were tested for normality using the Shapiro-Wilk test. Normally distributed variables were represented as mean (standard deviation) and comparisons were made using the Student’s *t*-test. Variables not normally distributed were represented by median (interquartile range) and comparisons were conducted using the Wilcoxon rank test. All analyses were performed using SAS 9.2 (Cary, NC, USA).

## Results

**Figure 1:**
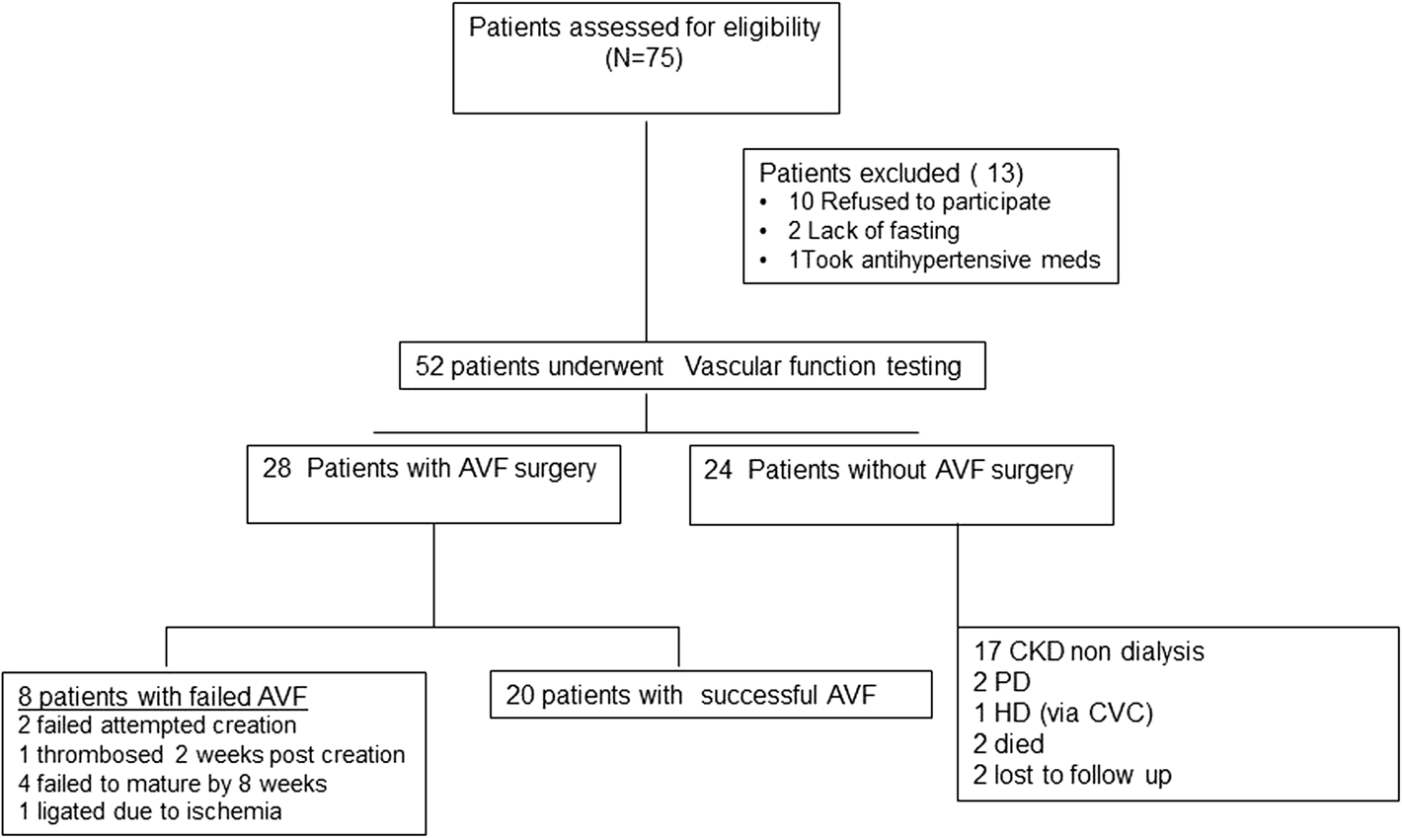
**Patient Flow Diagram.** Description of patient flow from start of study to completion.

We recruited 52 stage IV/V CKD patients to the study (37 men, 15 women) from June 1, 2006 until June 1, 2010, of whom 28 underwent AVF surgery and are the focus of this study (Figure 1). Patients were followed for one year after study entry to allow for dialysis initiation and functional assessment of the AVF (until June 1, 2011). Table 1 shows the baseline characteristics for the entire cohort as well as the 28 who underwent AVF surgery. The AVF patient population was older (median age 72), mostly males with a median eGFR of 15 ml/min/1.73 m [2] who had controlled blood pressure (median BP 135/78) with significant proteinuria (median protein to creatinine ratio of 0.36 corresponding to over 3 grams of protein/day) (Table 1). The majority of the study subjects had co-morbidities of: hypertension (100%), diabetes (56%), dyslipidemia (62%) and CAD (50%), reflective of a typical CKD population (Table 1). The mean time from AVF creation until dialysis initiation for this cohort was 12.9 ± 13 months.

**Table 1:** **Patient characteristics**

**Variable**	**AVF creation (%), n = 28**	**No AVF creation (%), n = 24**	**Entire cohort (n = 52)**
Age	72 (62-78)	71 (51-77)	71.5 (59.5 - 77.5)
Gender: Male	21 (75%)	16 (67%)	37 (71%)
Etiology of CKD			
Diabetes mellitus	8 (29%)	7 (29%)	15 (29%)
Hypertension	9 (32%)	5 (21%)	14 (27%)
Glomerulonephritis	1 (4%)	3 (13%)	4 (8%)
Obstruction	1 (4%)	2 (8%)	3 (6%)
Other	9 (32%)	7 (29%)	16 (31%)
Diabetes mellitus	14 (56%)	14 (58%)	28 (57%)
Hypertension	27 (100%)	22 (92%)	49 (96%)
Coronary artery disease, CAD	5 (50%)	5 (45%)	10 (48%)
Family history of CAD	1 (9%)	2 (18%)	3 (14%)
Smoker	4 (15%)	3 (13%)	7 (14%)
Peripheral vascular disease	3 (27%)	3 (27%)	6 (27%)
Dyslipidemia	8 (62%)	7 (58%)	15 (60%)
eGFR, ml/min/1.7 m^2^	15 (12-18)	24 (19-31)	17.5 (13 - 25)
Laboratory variables			
Hemoglobin, g/L	121 ± 14	121 ± 16)	121 (15)
Creatinine, umol/L	343 ± 88	231 ± 57)	291.54 ± 93.54
Urine microalbumin: creatinine ratio	285 (80-377)	15 (9-111)	95.7 (11.3 - 295)
Urine Protein: Cr ratio	0.36 (0.10-0.49)	0.03 (0.02-0.14)	0.11 (0.03 - 0.38)
Systolic blood/diastolic pressure left arm	135 (127-153)/78(70-88)	125 (115-148)/75 (68-83)	131.5 (119 - 149)/76 (69 – 85)
Mean arterial pressure	94 (89-106)	93 (84-101)	94 (87.67 - 104)
Beta blocker	8 (29)	3 (12)	11 (21)
Statin	9 (32)	5 (21)	14 (27)

### Fistula success

Of the 28 patients who underwent AVF surgery, 20 were successful and led to a mature AVF at 8 weeks. Amongst the 20 mature AVF, 18 were in use at HD start. The time from AVF creation until HD start was 11 ± 9.5 months for these patients. One patient had not yet started HD and another died before HD initiation. Of the unsuccessful AVF (8/28, 30%): 2 failed attempted AVF creation, 2 thrombosed post surgery, 4 failed to mature, and 1 was ligated at 6 weeks due to complications of steal. We did not find any significant differences in patient characteristics between those with a successful or unsuccessful AVF. The eGFR at the time of AVF creation (15 ml/min) and the degree of albuminuria (approximately 2.8 g albumin/day) was similar between the two groups of patients. Although not statistically significant the patients with successful AVF were mostly male (80% vs 63% in unsuccessful AVF, P = 0.33) and younger (71 vs 75 years, P = 0.54). The proportion of patients with diabetes and hypertension was similar amongst patients with successful and unsuccessful AVF.

The vascular function parameters are depicted in Table 2. The median FMD of 5.7 ± 4.3% in our study patients is lower than what has been reported in the general population [6]. The PAT was also low (1.73 ± 0.5) and similar to what has been reported for patients with CAD [16].

**Table 2:** **Vascular Function Tests amongst the AVF cohort**

**Variable**	**Overall**	**AVF outcome**		**P-value**
		**Unsuccessful n = 8**	**Successful n = 20**	
PWV, mean ± SD, m/s left arm	8.4 ± 1.7	8.2 ± 2.2	8.5 ± 1.5	0.70
Peripheral arterial tonometry, mean ± SD	1.73 (0.50)	1.41 (0.24)	1.87 (0.52)	**0.03**
Brachial artery diameter baseline; mean ± SD mm	4.7 ± 0.8	4.5 ± 0.8	4.8 ± 0.8	0.41
Velocity time integral baseline; mean ± SD, ml	15.3 (4.9)	16.4 (6.7)	14.9 (4.1)	0.47
Brachial artery– flow baseline; mean ± SD, ml/min	191 (92)	176 (92)	197 (94)	0.60
Brachial artery diameter with reactive hyperemia; mean ± SD mm	5.1 ± 0.8	4.9 ± 0.8	5.1 ± 0.9	0.58
Velocity time integral with reactive hyperemia; mean ± SD, ml	63 (33)	80 (50)	57 (23)	0.10
Brachial artery flow, with reactive hyperemia; mean ± SD, ml/min	838 (458)	884 (408)	818 (486)	0.74
Flow Mediated Dilation; mean ± SD, %	5.7 (4.3)	7.7 (5.9)	4.9 (3.3)	0.12

When comparing the patients with successful AVF to those with unsuccessful AVF we found very similar values in both groups for the FMD, PWV and VTI (Table 2). The PAT values, however, were higher for the patients with a successful AVF (1.87 ± 0.52) than those with failed AVF (1.41 ± 0.24 p = 0.03). The analysis was repeated with the ligated AVF included in the successful category (instead of unsuccessful as reported above) and all results remained similar (data not shown).

## Discussion

In this exploratory study we found that it is feasible to obtain vascular health parameters in a cohort of CKD patients prior to AVF creation. Our preliminary findings suggest that microvascular endothelial function as measured using PAT may be associated with subsequent successful AVF maturation and function. Given our small sample size we are unable to find any associations between the vascular health parameters of PWV, FMD or VTI and subsequent AVF success.

PAT is an accepted measure of microvascular endothelial function in the general population and those with cardiovascular disease and is known to be markedly decreased in individuals with CAD [17]. To our knowledge PAT has not been previously tested in the CKD population nor has it been used as a predictor of AVF maturation or success.

PAT is predominantly a measure of the peripheral small vessel function and is in part NO dependent. However, the stimuli that prompts the microvasculature to dilate to shear stress likely depends on other mediators such as prostaglandins, adenosine, potassium- ATP channels and endothelial derived hyperpolarizing factor [18]). The other marker of microvascular endothelial function, VTI was recently found to correlate with PAT, although this association is weak [19]. Given that VTI is measured using the brachial artery it may be that PAT and VTI measure two different microvascular beds and that the peripheral small vessel function is a better determinant of AVF success.

Hyperemic velocity or VTI has been suggested to be a better predictor of cardiac outcomes than FMD [6,20] because it is in fact the stimuli for FMD. The hyperemic velocity reflects both the endothelial function (the ability to release NO) and the endothelial structure (the ability to respond to hyperemia). In this study our CKD patients had a blunted reactive hyperemia response as compared to normal subjects [6] but this vascular marker was not predictive of subsequent AVF success. We are not aware of other studies exploring the VTI in CKD patients although there are reports of abnormal endothelial health [21].

Our study patients had impaired FMD with a median value of 5.0% (3-9) but we did not find any difference between the patients with successful and unsuccessful AVF. The finding of impaired endothelial function in this CKD cohort is similar to others have shown using both venous occlusion plethysmography techniques [12], and FMD [22]. To date, there are two other studies [23,24] that explore the relationship between FMD and AVF creation. Owens et al [23] determined FMD amongst 25 CKD patients prior to AVF creation and found very similar values to ours (5.8 ± 1.0%). They explored the correlation between baseline FMD and subsequent change in the diameter of the artery and vein at 3 months after AVF creation and found a positive correlation. Unlike in our study, they did not perform a maturation or functional assessment of the AVF and thus whether these changes in vascular diameter correlated to maturation or function is unknown. In our study we performed both a maturation assessment as well as a functional assessment at the time of dialysis start and all of our AVF that we had deemed mature were indeed functional at the time of dialysis initiation. We did not record the final diameter of the AVF but it is unlikely that we would have found a significant correlation given that our definition of mature AVF included a diameters ≥ 5 mm in accordance with the standard definition of mature AVF.

Genek et al [24] performed radial and brachial artery measurements, peak systolic blood flow rate and FMD prior to AVF creation. AVF assesments were made at 48 h post op and at day 30. Similar to our study, they did find any associations with FMD and subsequent AVF success but their period of follow up was relatively short.

PWV can decrease with the creation [25,26] of an AVF but it has not been previously explored as a predictor of AVF success. We did not find an association between underlying arterial stiffness in the periphery and subsequent AVF outcome. This probably reflects that regardless of the underlying stiffness of the vasculature, the more important aspect is the ability of the artery and vein to dilate and maintain the increase in blood flow required by successful AVF creation.

Maturation of an AVF is a complex process and not well understood. The factors that lead to a mature and functional AVF include: technical factors such as surgical expertise, location and angle of anastomosis [27], vascular health factors (diameter of vessels [28], endothelial function [23]) and patient factors such as elevated blood viscosity [29] leading to thrombosis. It is likely that one of the most important determinants of AVF maturation is the ability of the inflow artery and the outflow vein to respond to the increased blood flow that occurs upon anastomosis of the artery and vein. A healthy response to the anastomosis is an increase in blood flow and corresponding increase in shear stress [15] which stimulates the endothelial cells to release NO and other vasodilatory substances [30]. Many studies [26,31,32] have shown that there is first an immediate increase in blood flow upon anastomosis of artery to vein and subsequently the vein diameter increases. It is possible that the microvascular function, specifically the ability to dilate in response to the altered shear stress is a predictor of AVF success.

Studies are conflicting regarding the factors that are associated with AVF success. Most studies show that age [33], CAD and PVD are associated with a lower likelihood of a successful AVF [34,35]. However, diabetes [36,37] and female gender [38] are also associated with AVF failure in some but not all studies. Age, gender, DM and CAD were not predictive of AVF outcome in our cohort but this is likely due to our small sample size. In addition, within each of these patient factors there may be considerable variation in the underlying endothelial health and perhaps a better predictor is something that can more accurately reflect the endothelial function and structure such as PAT.

In this study we examined the response of the endothelium to the ischemic stress of brachial artery occlusion and measured the subsequent changes in diameter of artery and the blood flow velocity. Eight weeks after AVF creation successful AVF dilated to 5 mm or greater and they had a higher fingertip volume in response to reactive hyperemia. Since AVF maturation is dependent on endothelial function and the ability to respond to shear stress it seems logical that markers of endothelial function such as VTI and FMD may be predictive of maturation abilities. In our study however, we only showed an association with PAT and not VTI or FMD.

The main limitation of this study is the small sample size and the observational nature of the study. As pilot data these findings are intriguing and should be pursued in a larger study to determine if measures of microvascular endothelial function (PAT) predict successful AVF maturation. In addition, our patient population is predominantly male which limits the generalizability; it may be that there is some selection bias on the part of the nephrologist to refer females for AVF assessment. Furthermore, this CKD population was selected from individuals who are attending multidisciplinary Kidney Function Clinics which may limit the generalizability of the findings as these patients may represent a healthier patient population than those not followed at Kidney Function Clinics.

## Conclusions

The ability to have a reliable predictor of AVF success would be useful in the management of the pre-dialysis CKD patient. In individuals with poor prognostic factors for AVF success, conversations regarding the option of peritoneal dialysis could be reinforced or alternate choices of vascular access could be reviewed. Currently there are no established tools for predictors of AVF outcome, however, microvascular endothelial function as determined using PAT may be a useful tool which requires further exploration with a larger sample size.

## Abbreviations

AVF: Arteriovenous fistula
CAD: Coronary artery disease
CHF: Congestive heart failure
CKD: Chronic kidney disease
CVA: Cerebrovascular disease
DM: Diabetes mellitus
eGFR: Estimated GFR
FMD: Flow mediated dilation
HD: Hemodialysis
Hs CRP: High sensitivity C reactive protein
NO: Nitric oxide
PAT: Peripheral arterial tonometry
PTH: Parathyroid hormone
PVD: Peripheral vascular disease
PWV: Pulse wave velocity
VA: Vascular access
VTI: velocity time integral
